# A retrospective analysis of changes in distant and breast cancer related disease-free survival events in adjuvant breast cancer trials over time

**DOI:** 10.1038/s41598-022-09949-5

**Published:** 2022-04-15

**Authors:** Brooke E. Wilson, Alexandra Desnoyers, Laith Al-Showbaki, Michelle B. Nadler, Eitan Amir

**Affiliations:** 1 Division of Medical Oncology and Hematology, Department of Medicine, Princess Margaret Cancer Centre and the University of Toronto, 700 University Ave, 700U, 7W305, Toronto, ON M5G 2M9 Canada; 2 University of New South Wales, Kensington, NSW Australia

**Keywords:** Cancer, Oncology

## Abstract

Disease-free survival (DFS) comprises both breast cancer and non-breast cancer events. DFS has not been validated as a surrogate endpoint for overall survival (OS) in most breast cancer subtypes. We assessed changes to the type of events contributing to DFS over time. We identified adjuvant studies in breast cancer (BC) from 2000 to 2020 where the endpoint was DFS. We examined change in distant DFS events and the BC-related DFS using univariable and multivariable linear regression. Data were reported quantitatively using the Burnand criteria irrespective of statistical significance. We included 84 studies (88 cohorts), comprising 212,191 participants, 41,604 DFS events and 23,205 distant DFS events. The DFS event rate/100 participants/year has declined modestly over time (ß − 0.34, p = 0.001). Start year was negatively associated with distant DFS events (ß − 0.58, p < 0.0001); however, the effect was lost after adjusting for follow-up time (ß − 0.18, p = 0.096). The average number of BC-related events/100 participants/year also declined over time (ß − 0.28, p = 0.009). In multivariable analysis, start year and ER expression were quantitatively associated with distant DFS events and BC-related DFS events. DFS events have declined over time driven by a reduction in BC related events. As DFS events are increasingly defined by non-BC events, there will be limited surrogacy between DFS and OS.

## Introduction

Over time, regulatory approvals based on intermediate endpoints in oncology have become more common^1^. Although overall survival (OS) remains the gold standard endpoint to assess the efficacy of a treatment for women with breast cancer, disease free survival (DFS), invasive-DFS (iDFS), event-free survival (EFS) and relapse-free survival (RFS) are often preferred endpoints in adjuvant breast cancer trials. DFS is a composite endpoint typically defined as time until local recurrence, contralateral recurrence (and/or new primary breast cancers), distant disease, secondary cancers or death from any cause. The broad definition of DFS can include both invasive and in-situ recurrences, whereas iDFS excludes ductal carcinoma in situ (DCIS) as an event^2^. EFS is defined as time to progression of disease that precludes surgery, local or distant recurrence and death due to any cause and is used more commonly in neoadjuvant trials. RFS includes any recurrence (local, regional or distant) or death from any cause, but does not include new cancer(s). All of these endpoints require a shorter follow-up time and smaller sample size due to the higher number of events, and can expedite the time required for trial completion compared to using OS as the primary endpoint^3^. This can lead to more rapid market approval, reducing the time needed for a drug to reach patients.

Although DFS is a recognised endpoint by regulators, it has not been validated as a surrogate for OS in early breast cancer^4^, with the exception of studies in HER2 positive disease^5^. In other words, for HER2-negative breast cancers, adjuvant treatments that prolong DFS may not translate to improved OS at a trial level and DFS may not capture the net effect of treatment on OS. If DFS events are driven predominantly by local or contralateral recurrences that can be treated with curative intent, secondary cancers or deaths from causes other than breast cancer, the lack of association with OS both at trial levels and at treatment level is unsurprising. Furthermore, if DFS does not translate into improved OS, the utility of adjuvant treatments based on improved DFS alone should be considered carefully against the costs and potential toxicities.

The objective of this study is to examine how the determinants of DFS in adjuvant breast cancer trials have changed over time. We hypothesize that over time, distant and BC-related DFS events have declined. This would further decrease the surrogacy of DFS and OS over time. Specifically, we aim to examine: (1) changes in total number of DFS events; (2) changes in distant DFS events; and (3) changes in breast-cancer related events, each as a proportion of the total population randomized over time.

## Methods

### Study eligibility and identification

In March 2021, we searched for large adjuvant breast cancer trials in MEDLINE (host: Pubmed). We supplemented this search by reviewing citation lists from publications by the Early Breast Cancer Trialists Collaborative Group. The search strategy is outlined in Appendix 1. Trials meeting the following criteria were included: publication between 2000 and 2020, English language, randomized phase III studies, examining human patients with early breast cancer treated with either adjuvant chemotherapy, endocrine therapy, targeted treatments (e.g. HER2, CDK4/6 inhibitors), or bisphosphonates. Only studies where DFS or iDFS was the primary or secondary endpoint were included, while RFS and time to tumour recurrence endpoints were excluded. Adjuvant studies with EFS as the primary or secondary endpoint were also included if the definition was time from randomization to locoregional or distant recurrence, new breast primary, or death form any causes. A full list of inclusion and exclusion criteria can be found in Supplementary Table 1. For studies with multiple publications over time, the publication with the longest follow-up time was used. Titles and abstracts were screened using Covidence by one author (BW) and data extraction was performed by 2 authors (BW and AD).

### Data extraction

For included studies, the following data were extracted: year of publication, trial start and stop dates, median follow-up (FU) time, control and intervention arm treatments, number of participants in each arm, population characteristics (median age, and proportion node positive, HR positive, grade 3, premenopausal), primary and secondary endpoints with associated HR and 95% CI. We reviewed the primary publication and the supplemental appendices to extract the types of DFS/iDFS/EFS events, which were grouped into distant breast cancer recurrence, locoregional recurrence, contralateral recurrence, other cancer, death without breast cancer recurrence, unknown and DCIS. We then calculated the pooled BC-related events (distant recurrence, locoregional recurrence, contralateral recurrence) and the pooled non-BC related events (death other causes, second primary cancers, other and unknown) for each study.

### Statistical analyses

We calculated the total DFS event rate per year per 100 participants as a proportion of the total population randomized as {([all DFS events/total population randomized]/[FU time in years]) × 100}, the distant DFS event rate per year per 100 participants as a proportion of the total population randomized by study as {([distant DFS events/total population randomized]/[FU time in years]) × 100}, and the BC related DFS event rate per year per 100 participants as a proportion of all participants randomized as {([BC related DFS events/total population randomized]/[FU time in years]) × 100}.

To examine changes in DFS event type over time, we performed univariable linear regression weighted by trial sample size exploring the association between trial start year on (i) DFS event type as a proportion of all participants randomized, and (ii) DFS event type/100 participants randomized/year. We also performed univariable regression to examine the association between BC related and non-BC related events as a proportion of all participants randomized and study follow-up time. Finally, we explored the association between distant DFS events and BC related DFS events/100 participants/year and proportion of women premenopausal, proportion node positive, proportion grade 3 disease, and proportion ER positive disease.

Multivariable linear regression weighted by sample size was performed to examine the association between trial start year and (i) distant DFS events as a proportion of total sample size and (ii) BC related DFS events as a proportion of participants randomized, adjusting for FU time, proportion women premenopausal, proportion node positive and proportion ER positive disease. The proportion with grade 3 disease was not included in the primary multivariate models due to the amount of missing data but was explored through sensitivity analysis.

We present the standardized coefficients (ß), and assessed quantitative significance using methods described by Burnand et al.^6^, where ≥ 0.28 is considered quantitatively significant irrespective of statistical significance. All analyses were performed using STATA version 12.0 (StataCorps LP, College Station, TX, USA). Statistical significance was defined as p < 0.05. Corrections for multiplicity were not applied in this hypothesis generating study.

## Results

We identified 1204 unique articles using our search strategy, and an additional 24 were identified through review of citation lists. We excluded 981 on title, and a further 82 after abstract review. We evaluated 165 full texts and excluded a further 73 trials. The final analysis included 84 studies, of which 4 had data presented in multiple cohorts, resulting in 88 cohorts eligible for analysis (Supplementary Fig. 1).

The majority of cohorts included mixed breast cancer histology (39.8%), while 36.4% included only ER positive patients, 13.6% only HER2 positive patients, 5.7% HER2 negative histology and 4.6% only triple negative breast cancer patients (Supplementary Table 2). The most common trial interventions were chemotherapy vs chemotherapy (37.5%), followed by different endocrine therapy strategies (25%). The median follow-up duration was 70.8 months (range 15.5–360 months). Across all included studies, 212,191 participants were evaluable for DFS. There were 41,604 DFS events, of which 23,205 were distant DFS events. The median number of DFS events per cohort was 306 (range 27–1975), and the median number of distant DFS events per cohort was 184.5 (range 11–1025). The majority of included studies used DFS or iDFS as the primary or secondary endpoint (94.5%) (Table 1).

**Table 1:** DFS endpoint characteristics.

	N = 88 cohorts (84 unique studies)
Total number of participants included in DFS analysis across all studies	212,191
Total DFS events, all studies	41,604
Total distant DFS events, all studies	23,205
Median number of participants contributing to DFS endpoint (range)	1629 (58^a^–9366)
Median number of DFS events per study (range)	306 (27–1975)
Median number of distant DFS events per study (range)	184.5 (11–1025)
Median % distant events by study	57.0 (21.1–83.3%)
**DFS-type endpoint**	
iDFS	9 (10.2%)
DFS	74 (84.1%)
EFS	5 (5.7%)

^a^Although we excluded studies with less than 100 participants, this study was split into 2 cohorts for the analysis, but the total number of participants in the study was above 100.

This table shows the DFS characteristics of the 88 cohorts included in this analysis. *DFS* disease free survival, *iDFS* invasive disease free survival, *EFS* event free survival.

### Changes in DFS events and median follow-up duration

As a proportion of all randomized participants, there was a quantitatively significant rise in distant DFS events (ß 0.63), locoregional events (ß 0.31), contralateral events (ß 0.30), deaths (ß 0.60), BC-related events (ß 0.60) and non-BC related (ß 0.64) events as median FU duration increased. Distant DFS events as a proportion of all DFS events was relatively constant at approximately 58% (ß − 0.03) regardless of the median FU duration. Similarly, there was no significant change in the proportion of DFS events that are contralateral events, locoregional relapses, deaths, or unknown/other and median FU duration. BC related events as a proportion of all DFS events declined slightly (ß − 0.16) with increasing median FU duration, while non-BC related events increased slightly (ß 0.16), though neither change was quantitatively significant (Supplementary Fig. 3A-N).

A single outlier study with 360 months of follow-up data was excluded from the above analyses. Sensitivity analysis including this study did not demonstrate any changes in the quantitatively significant variables (Supplementary Table 5).

### DFS events type as a proportion of the total population randomized and trial start year, crude and adjusted analyses

**Figure 1:**
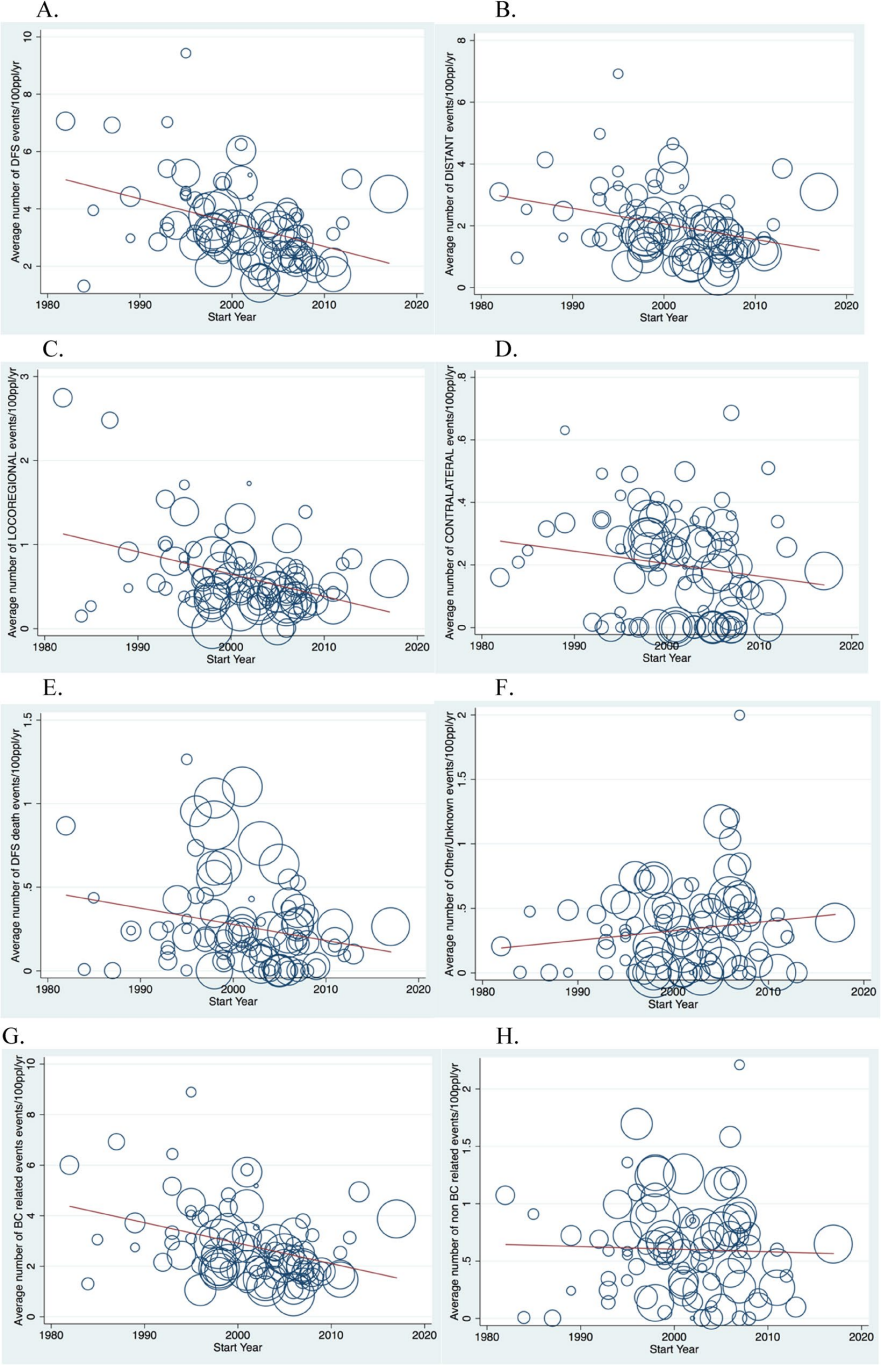
(**A**–**H**) Average number of DFS events, by type of event, per 100 ppl randomized per year of median follow-up (n = 88).

There was a negative association between the proportion of randomized participants with any DFS events and trial start year (ß − 0.68, p < 0.0001) (Supplementary Fig. 2A), with DFS events ranging from 35 to 70% in studies initiated prior to 1990, as compared to between 5.8 and 19.5% for those initiated after 2010. After adjusting for median follow-up duration, DFS events/100 participants/year has declined modestly over time (ß − 0.34, p = 0.001) (Fig. 1A).

There was a negative association between the proportion of randomized participants with a distant DFS events and trial start year (ß − 0.58, p < 0.0001) (Supplementary Fig. 2B), with distant DFS events ranging from 19 and 31% in studies initiated prior to 1990, compared to between 4 and 13% in studies initiated after 2010. However, quantitative significance between distant DFS and start year was lost after adjusting for FU duration in univariable analysis (ß − 0.18, p = 0.096) (Fig. 1B).

The proportion of randomized participants with a locoregional DFS event also declined over time (ß − 0.55, p < 0.0001) (Supplementary Fig. 2C), and this remained quantitatively significant after adjusting for FU duration (ß − 0.36, p = 0.001) (Fig. 1C). Contralateral events were uncommon across all studies, and the association with start year after adjusting for FU time did not meet the threshold for quantitative significance despite statistical significance (ß − 0.24, p = 0.023) (Fig. 1D). The proportion of randomized participants with deaths as first DFS event was also low across all studies and was negatively associated with trial start year in crude analysis (ß − 0.37, p < 0.0001) (Supplementary Fig. 2E), and after adjusting for FU time (ß − 0.29, p = 0.006) (Fig. 1E). There was no association other/unknown DFS events and trial start year on crude (ß − 0.11, p = 0.3) (Supplementary Fig. 2F) or adjusted analysis (ß 0.08, p = 0.44) (Fig. 1F).

In unadjusted analysis, there was a negative association between trial start year and the proportion of randomized participants with BC-related events (ß − 0.64, p < 0.0001) (Supplementary Fig. 2G) and non-BC related events (ß − 0.37, p < 0.001) (Supplementary Fig. 2H). Adjusting for FU duration, BC related events/100 participants/year declined by trial start year (ß − 0.28, p = 0.009) (Fig. 1G), while there was no significant change in non-BC related events/100 participants/year (ß − 0.17, p = 0.11) (Fig. 1H).

### Association between the distant and BC related DFS event rate and baseline study variables

**Figure 2:**
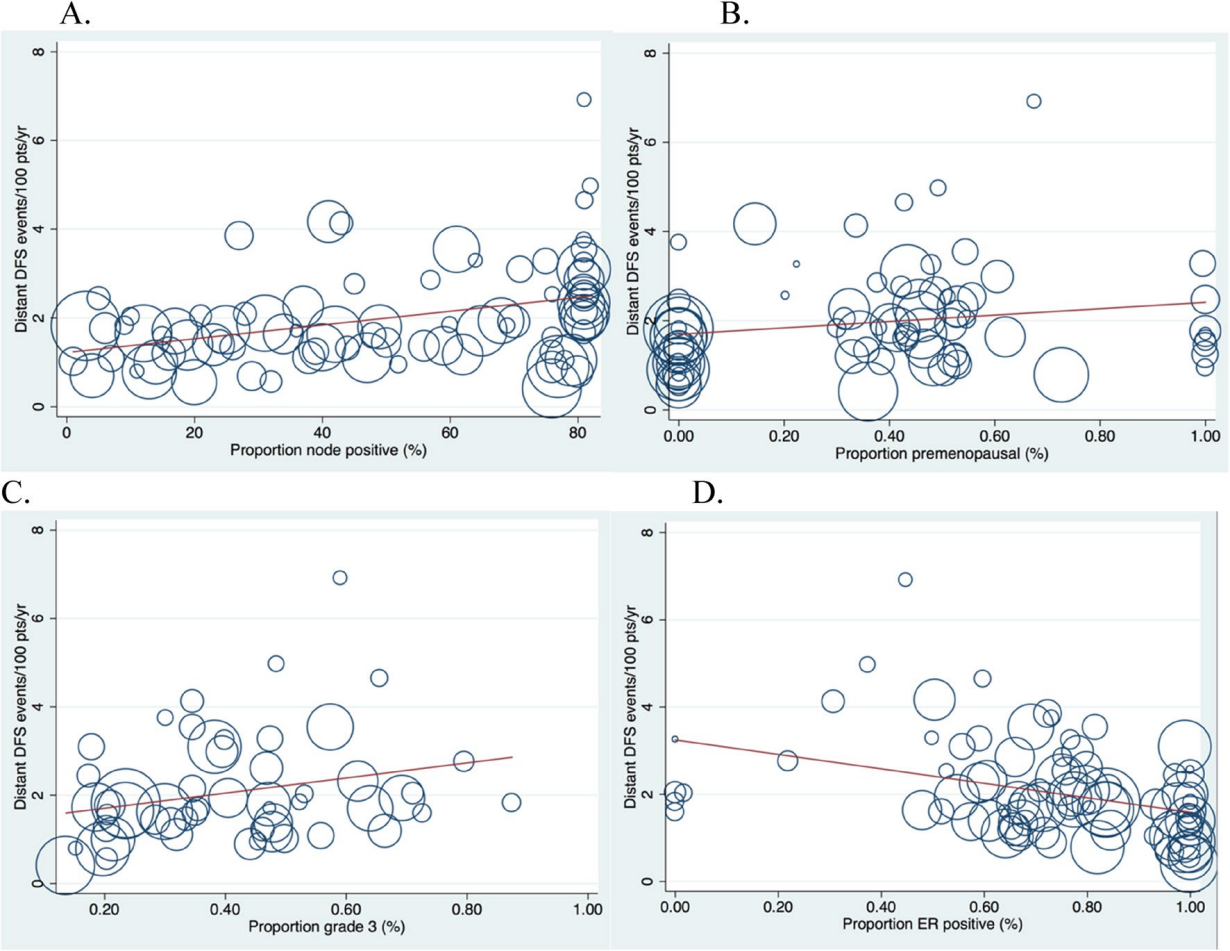
(**A**–**D**) Distant DFS event rate/per 100 participants/year as a percentage of all participants randomized, stratified by co-variates. (**A**) % of participants node positive (n = 84). (**B**) % pre menopausal (n = 75). (**C**) % grade 3 (n = 53). (**D**) % ER positive participants (n = 84).

We found a positive association between distant DFS events/100 participants/year and the proportion with grade 3 disease (ß 0.35, p = 0.01), and a weak positive association with proportion of node positive participants (ß 0.27, p = 0.013), and the proportion pre-menopausal (ß 0.22, p = 0.054) (Fig. 2A–C). There was a negative association between the proportion with ER positive disease and distant DFS events/100 participants/year (ß − 0.40, p < 0.0001, Fig. 2D).

**Figure 3:**
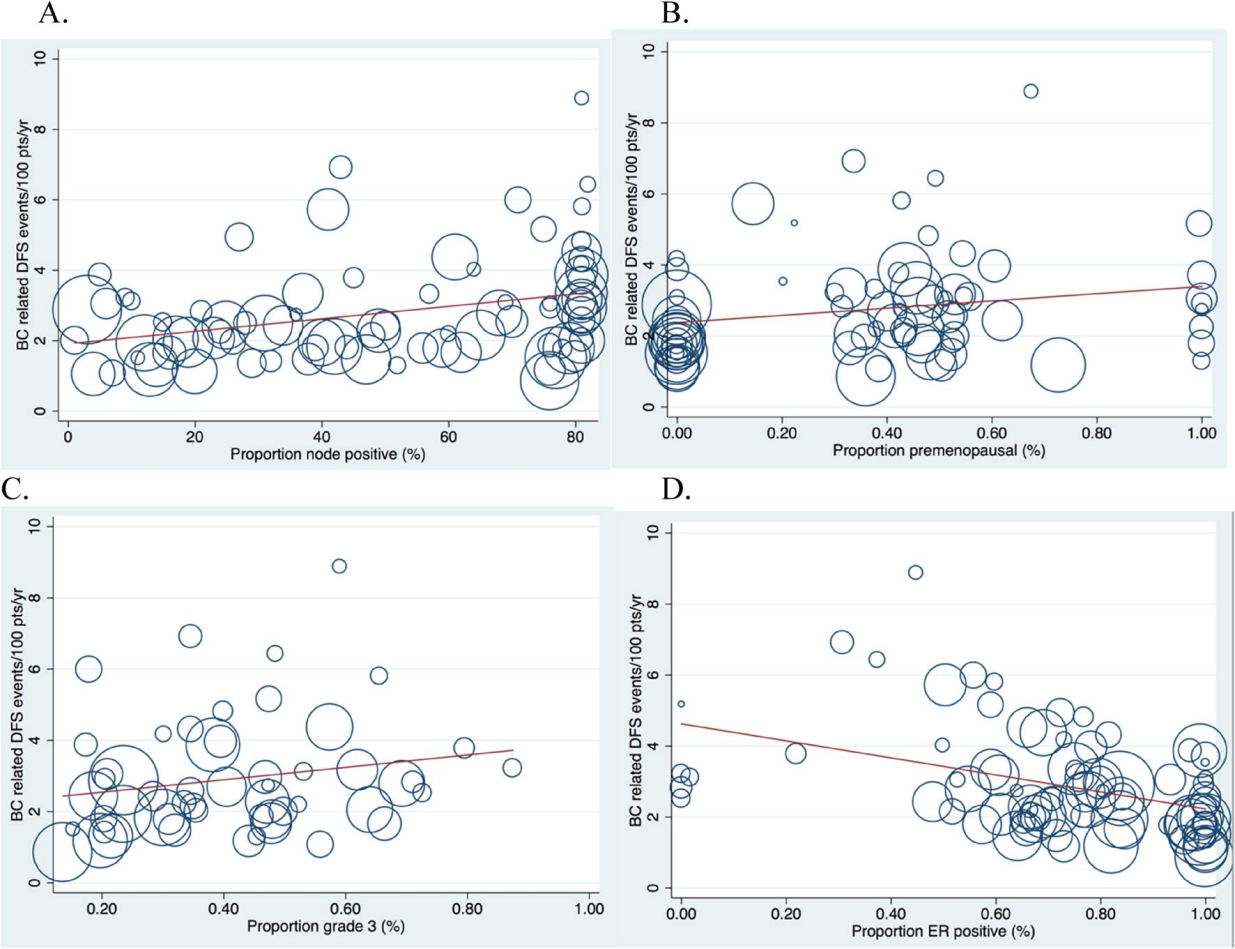
(**A**–**D**) BC related DFS event rate/per 100 participants/year as a percentage of all participants randomized, stratified by co-variates. (**A**) % of participants node positive (n = 84). (**B**) % pre menopausal (n = 75). (**C**) % grade 3 (n = 53). (**D**) % ER positive participants (n = 84).

The rate of BC related DFS events/100 ppl/year was positively associated with the proportion node positive disease (ß 0.24, p = 0.026), proportion premenopausal (ß 0.24, p = 0.04) and proportion grade 3 (ß 0.27, p = 0.048), although none met quantitative significance. There was a quantitatively significant negative association between BC related DFS events/100 participants/year and proportion ER positive (ß − 0.43, p < 0.0001, Fig. 3A–D).

### Multivariable analysis

In multivariable analysis, the proportion of randomized participants with a distant DFS event was negatively associated with trial start year and the proportion with ER positive disease and was positively associated with median FU time (Table 2). The proportion of randomized participants with a BC related DFS events was negatively associated with trial start year and the proportion with ER positive disease and was positively associated with median FU time (Table 3). Sensitivity analysis in the subgroup of studies reporting the proportion with grade 3 disease (n = 45) can be found in Supplementary Tables 3 and 4.

**Table 2:** Multivariate linear regression for distant DFS events as a percentage of total participants randomized, weighted by sample size (n = 68).

Variable	B-coefficient	Standardised ß-coefficient	P
Start year	− 0.41	− 0.34	< 0.0001
Median FU time (months)	0.10	0.48	< 0.0001
% node positive	0.06	0.27	0.001
% ER positive	− 11.9	− 0.34	< 0.0001
% premenopausal	0.66	0.028	0.73

R^2^ 0.68.

*FU* follow-up, *ER* estrogen receptor.

**Table 3:** Multivariate linear regression for BC related DFS events as a percentage of total participants randomized, weighted by sample size (n = 68).

Variable	B-coefficient	Standardised ß-coefficient	P
Start year	− 0.67	− 0.42	< 0.0001
Median FU time (months)	0.11	0.40	< 0.0001
% node positive	0.07	0.23	0.002
% ER positive	− 15.9	− 0.33	< 0.0001
% premenopausal	3.44	0.11	0.15

R^2^ = 0.71.

*FU* follow-up, *ER* estrogen receptor.

## Discussion

Evidence from single studies suggests that with longer follow-up, DFS is less commonly due to distant recurrence, and more commonly defined by contralateral recurrence or non-breast cancer related deaths^7^. To our knowledge, this is the first study to examine the change in the types of events contributing to DFS endpoints over time in a large cohort of adjuvant breast cancer trials. We demonstrate that distant DFS events as a proportion of all participants randomized have declined over time, from between 19 and 31% in studies initiated prior to 1990 compared to between 4 and 13% in studies initiated after 2010. After adjusting for other variables including duration of follow-up, trial start year was the strongest predictor of distant and BC related events as a proportion of all participants randomized. In contrast, the proportion of all participants randomized without any DFS event has increased over time, while non-BC-related events as a proportion of all participants randomized have remained relatively constant.

If DFS events are driven increasingly by non-distant recurrence or non-BC related events, the net effect of breast cancer therapy on DFS may not translate to OS and this will result in limited surrogacy between DFS and OS for adjuvant treatments for breast cancer. For example, in TAILORx, only 24% of iDFS events were due to distant disease recurrence, while 28% were related to locoregional recurrence or contralateral breast cancer and 48% were unrelated to breast cancer (other second primary cancers or death from other causes)^8^. As such, the impact of adjuvant chemotherapy on DFS is unlikely to translate into OS benefit in this trial.

Our results raise concerns regarding the approval of adjuvant breast cancer treatment based on the use of DFS as a surrogate endpoint for OS. Recently, the Food and Drug Administration has approved adjuvant breast cancer therapies based on small DFS gains^9^ which are unlikely to translate into any OS benefits. As an example, Neratinib was approved as adjuvant therapy for early HER2 + BC patients based on the ExteNET trial, which found only a 2.3% improvement in invasive DFS at 2 years^10^. Overpowering studies by enrolling large numbers of participants such that small differences in DFS are statistically but perhaps not clinically meaningful will lead to costly overtreatment of patients for minimal (if any) gains.

Alternative endpoints for large adjuvant breast trials should be considered. A composite of distant breast cancer recurrence or death might be a more robust surrogate for OS and should be explored. As a minimum, adjuvant trials in breast cancer must clearly present the types of events contributing to DFS to allow clinicians to better understand whether a treatment will meaningfully impact on a patient’s outcomes and long-term prognosis. Of the 165 studies identified for full text review, almost a third were excluded for failure to present the DFS events clearly. The exclusion of studies not clearly presenting these data could bias our results.

Several potential reasons for the observed decline in distant and BC-related events over time are proposed. First, the true underlying risk of breast cancer recurrence for patients enrolling in adjuvant breast cancer trials over time is likely to have decreased, due to earlier detection, improved surgical and radiation interventions, and improved systemic treatment options. Alternatively, the apparent fall in DFS events may have resulted from stage migration, whereby better diagnostic techniques have improved our ability to accurately classify the stage of disease^11^. For example, our ability to identify patients with small volume metastases through more sensitive imaging technologies has improved, such that the chance of enrolling a patient with occult metastases on an adjuvant trial has likely declined. Additionally, eligibility criteria for adjuvant breast cancer trials may have changed^12^, leading to the inclusion of participants with better prognosis in more recent studies. However, even after adjusting for the proportion of participants with node positive disease and grade 3 disease in multivariable models (which are recognised markers for the risk of breast cancer recurrence^13,14^), start year remained strongly associated with distant DFS events and BC related DFS events as a proportion of all participants randomized, with earlier studies having higher distant and BC related DFS event rates when compared to later studies. Unfortunately, due to variability in the reporting of tumour size, and the lack of complete reporting of additional potential confounders such as stage distribution, molecular characteristics and genomic risk, adjustments for other potential causes of improved outcomes over time were not possible. This residual confounding could affect the validity of our results.

In calculating the DFS events adjusted for FU time, we assume a linear association between the event rate and time. In reality there may be a non-linear association between the types of events over time, particularly for certain subtypes of breast cancer. Triple negative breast cancers have a relatively high rate of recurrence and death in the first 5 years following diagnosis^15^, as compared to ER positive breast cancers where approximately 50% of recurrences occur beyond the initial 5 years of FU^16^. Therefore, the assumption that events recur relatively constantly over time could lead us to overestimate the time-averaged event rate in more recent studies with shorter median follow-up, especially in studies with a high proportion of triple negative disease. However, this would be expected to underestimate the negative association between distant and BC-related DFS events over time.

## Conclusions

Over time, the number of distant and BC-related DFS events as a proportion of all participants randomized in adjuvant trials for breast cancer has declined, even after adjusting for baseline trial variables and median follow-up time. As a result, there will likely be diminishing surrogacy between DFS and OS over time. Providing treatments to patients with breast cancer in the adjuvant setting based on improved DFS which may not translate to improved OS may result in minimal gains and increased toxicity. The oncology community should reflect on the optimal endpoints for adjuvant breast cancer trials and ensure that components of composite endpoints such as DFS are reported accurately. This would maximise patient benefit and minimize harms and costs.

## Supplementary Information

Supplementary Information.

## Data Availability

The datasets used and/or analysed during the current study are available from the corresponding author on reasonable request.

## References

[1] Kay A (2012). Randomized controlled trials in the era of molecular oncology: Methodology, biomarkers, and end points. Ann. Oncol..

[2] Administration, F.A.D. *Clinical Trial Endpoints for the Approval of Cancer Drugs and Biologics Guidance for Industry*. https://www.fda.gov/media/71195/download (2018). (Accessed 12 January 2021).

[3] Chen EY (2019). Estimation of study time reduction using surrogate end points rather than overall survival in oncology clinical trials. JAMA Intern. Med..

[4] Gyawali B, Hey SP, Kesselheim AS (2020). Evaluating the evidence behind the surrogate measures included in the FDA's table of surrogate endpoints as supporting approval of cancer drugs. EClinicalMedicine.

[5] Saad ED (2019). Disease-free survival as a surrogate for overall survival in patients with HER2-positive, early breast cancer in trials of adjuvant trastuzumab for up to 1 year: A systematic review and meta-analysis. Lancet Oncol..

[6] Burnand B, Kernan WN, Feinstein AR (1990). Indexes and boundaries for “quantitative significance” in statistical decisions. J. Clin. Epidemiol..

[7] Algorashi I (2018). Evolution in sites of recurrence over time in breast cancer patients treated with adjuvant endocrine therapy. Cancer Treat. Rev..

[8] Sparano JA (2018). Adjuvant chemotherapy guided by a 21-gene expression assay in breast cancer. N. Engl. J. Med..

[9] FDA. *FDA approves neratinib for extended adjuvant treamtent of early stage HER2 positive breast cancer*. https://www.fda.gov/drugs/resources-information-approved-drugs/fda-approves-neratinib-extended-adjuvant-treatment-early-stage-her2-positive-breast-cancer (2018). (Accessed 12 January 2021).

[10] Martin M (2017). Neratinib after trastuzumab-based adjuvant therapy in HER2-positive breast cancer (ExteNET): 5-year analysis of a randomised, double-blind, placebo-controlled, phase 3 trial. Lancet Oncol..

[11] Polednak AP (2015). Increase in distant stage breast cancer incidence rates in US women aged 25–49 years, 2000–2011: The stage migration hypothesis. J. Cancer Epidemiol..

[12] Srikanthan A (2016). Evolution in the eligibility criteria of randomized controlled trials for systemic cancer therapies. Cancer Treat. Rev..

[13] Truong PT (2005). The prognostic significance of the percentage of positive/dissected axillary lymph nodes in breast cancer recurrence and survival in patients with one to three positive axillary lymph nodes. Cancer Interdiscip. Int. J. Am. Cancer Soc..

[14] Simpson JF (2000). Prognostic value of histologic grade and proliferative activity in axillary node–positive breast cancer: Results from the eastern cooperative oncology group companion study, est 4189. J. Clin. Oncol..

[15] Dent R (2007). Triple-negative breast cancer: Clinical features and patterns of recurrence. Clin. Cancer Res..

[16] Pan H (2017). 20-year risks of breast-cancer recurrence after stopping endocrine therapy at 5 years. N. Engl. J. Med..

