# Supplementary material for: A retrospective analysis of changes in distant and breast cancer related disease-free survival events in adjuvant breast cancer trials over time
Source: Sci Rep. 2022 Apr 15;12:6352. doi: 10.1038/s41598-022-09949-5 (PMC9012825; doi:10.1038/s41598-022-09949-5)
Supplement: Supplementary file 1 — Supplementary Information. [file 41598_2022_9949_MOESM1_ESM.docx]

**Supplemental Table 1: Inclusion and Exclusion Criteria**

| Inclusion Criteria | Exclusion Criteria |
| --- | --- |
| Studies published between 2000-2020 | Studies published prior to 2000 (although participants could have been enrolled prior to 2000) |
| Phase III | Phase I or II |
| Examining interventions in the adjuvant setting | Examining interventions in the metastatic or neoadjuvant setting |
| Studies comparing systemic therapy interventions (chemotherapy, endocrine therapy, targeted treatments), or the sequencing or delivery schedules of these treatments | Studies examining surgical or radiotherapy interventions, or the sequencing or delivery schedules of surgical and radiotherapy interventions |
| Primary or secondary outcome DFS, iDFS, EFS | Primary or secondary outcome of recurrence free survival or time to tumor progression |
| Break-down of the first contributing event for DFS endpoint provided (eg local, regional and distant events) | Studies examining supportive care interventions, correlative studies, sub-studies |
| Superiority or non-inferiority study designs | Studies with <100 participants randomized |
| Longest available follow-up period for any give study | Studies of biosimilars |
|  | Studies meeting criteria, but with later follow-up available |
|  | Studies where the DFS/EFS/RFS endpoints were not mutually exclusive |

**Supplemental Table 2: Characteristics of 88 included cohorts in DFS analysis**

|  | N=88 cohorts |
| --- | --- |
| Histology  ER +  HER2+  HER2-  TNBC  Mixed histology | 32 (36.4%)  12 (13.6%)  5 (5.7%)  4 (4.6%)  35 (39.8%) |
| Type of Intervention tested  Endo vs Endo  Chemo vs Endo  Chemo vs Chemo  Sequencing/duration  Chemo vs placebo  Endo vs placebo  Targeted vs placebo  Addition of bisphosphonate | 22 (25.0%)  3 (3.4%)  33 (37.5%)  6 (6.8%)  6 (6.8%)  7 (7.9%)  9 (10.2%)  2 (2.3%) |
| Median follow-up time, median (range) | 70.8 months  (15.5 months – 360 Months) |
| Start Year  1980-1990  1991-2000  2001-2005  2006-2010  2011-2020 | 6 (6.8%)  30 (34.1%)  25 (28.4%)  21 (23.9%)  6 (6.8%) |
| % ER positive  (n=84) | 75.5%  (0-100%) |
| % node positive  (n=84) | 51%  (1-82%) |
| % premenopausal  (n=75) | 40.9%  (0-100%) |
| Weighted median age  (n=60) | 51.5 years  (35.3 – 65.2 years) |
| Weighted proportion with grade 3 histology  (n=53) | 40.0%  (13.6 – 87.5%) |

Abbreviations: ER – estrogen receptor; Endo – endocrine; Chemo- chemotherapy; TNBC- triple negative breast cancer;

**Supplemental Table 3: Multivariate linear regression for Distant DFS events as a percentage of total participants randomized, weighted by sample size (n=45)**

| Variable | B-Coefficient | Standardised ß-Coefficient | P |
| --- | --- | --- | --- |
| Start year | -0.68 | -0.58 | <0.0001 |
| Median FU time (months) | 0.076 | 0.35 | 0.001 |
| Proportion node positive | 0.06 | 0.28 | 0.006 |
| Proportion ER positive | 0.28 | 0.008 | 0.95 |
| Proportion premenopausal | -1.6 | -0.07 | 0.47 |
| Proportion grade 3 | 16.6 | 0.40 | 0.008 |

R2 0.72

**Supplemental Table 4: Multivariate linear regression for BC related DFS events as a percentage of total participants randomized, weighted by sample size (n=45)**

| Variable | B-Coefficient | Standardised ß-Coefficient | P |
| --- | --- | --- | --- |
| Start year | -0.98 | -0.64 | <0.0001 |
| Median FU time (months) | 0.07 | 0.25 | 0.014 |
| Proportion node positive | 0.08 | 0.27 | 0.009 |
| Proportion ER positive | -4.8 | -0.11 | 0.45 |
| Proportion premenopausal | 1.09 | 0.04 | 0.70 |
| Proportion grade 3 | 14.0 | 0.26 | 0.078 |

R2=0.72

Abbreviations: FU – follow-up; ER – estrogen receptor;

**Supplemental Table 5: Sensitivity analysis for the association between DFS events and median follow-up time**

| **Variable** | **Standardized ß-Coefficient excluding study with FU time of 360 months** | **Standardized ß-Coefficient including study with FU time of 360 months** |
| --- | --- | --- |
| **Type of DFS event as a proportion of all participants randomized** | | |
| Distant events | 0.63 | 0.62 |
| Locoregional events | 0.31 | 0.29 |
| Contralateral events | 0.30 | 0.36 |
| Death | 0.60 | 0.51 |
| Other events | 0.25 | 0.19 |
| BC related events | 0.60 | 0.59 |
| Non-BC related events | 0.64 | 0.53 |
| **Type of DFS event as a proportion of all participants with a DFS event** | | |
| Distant events | -0.03 | 0.004 |
| Locoregional events | -0.22 | -0.22 |
| Contralateral events | -0.08 | -0.03 |
| Death | 0.24 | 0.19 |
| Other events | -0.02 | -0.04 |
| BC related events | -0.16 | -0.11 |
| Non-BC related events | 0.26 | 0.11 |

Abbreviations: BC – breast cancer; DFS – disease free survival; FU – follow-up

**Supplemental Figure 1: Schema for Study Inclusion**

Articles identified by search (after removal of duplicates)

n= 1,204

Studies Excluded on Title

(n=981)

Studies added after Manual Search or Oxford Overview Search

(n=24)

Number of Abstracts assessed

n=247

Excluded after abstract review (n=82)

38 duplicate data (earlier publication or subgroup

12 wrong intervention

17 wrong setting/population

9 wrong outcome

1 not more than 100 participants

2 wrong time window

Excluded after full text review (n=73)

No DFS/EFS endpoint (n=7)

RFS or time to tumour recurrence (n=10)

DFS breakdown not provided (n=49)

DFS endpoint not mutually exclusive (n=4)

Full text unavailable (n=6)

Duplicate data (n=5)

Included Studies (n=84)

(4 with 2 cohorts)

(N unique cohorts =88)

Full Text Reviews (n=165)

Supplemental Figures 2A-F: DFS event type over time as a proportion of all participants randomized (unadjusted, n=88)

1. B.


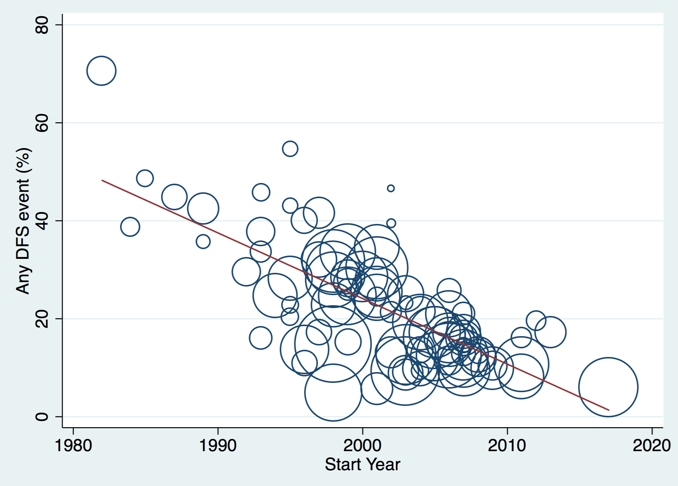

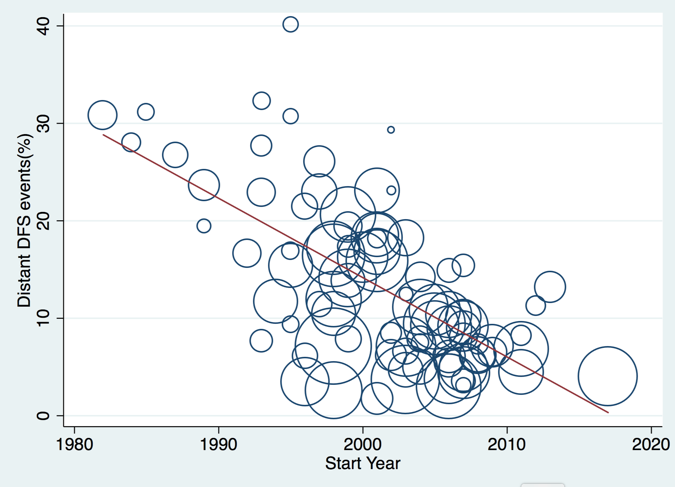


C. D.


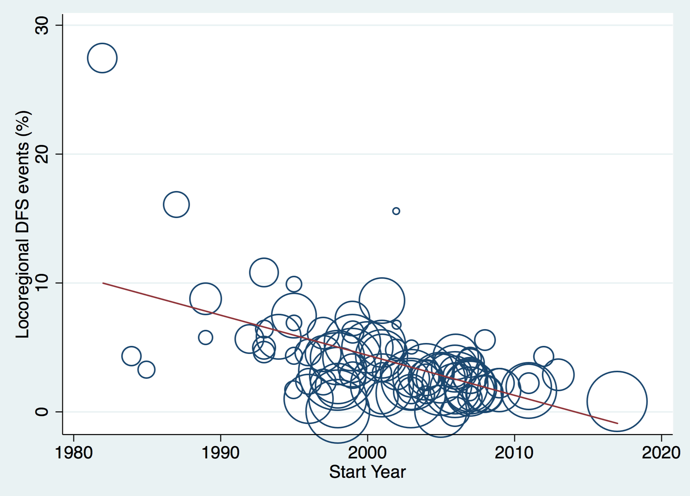

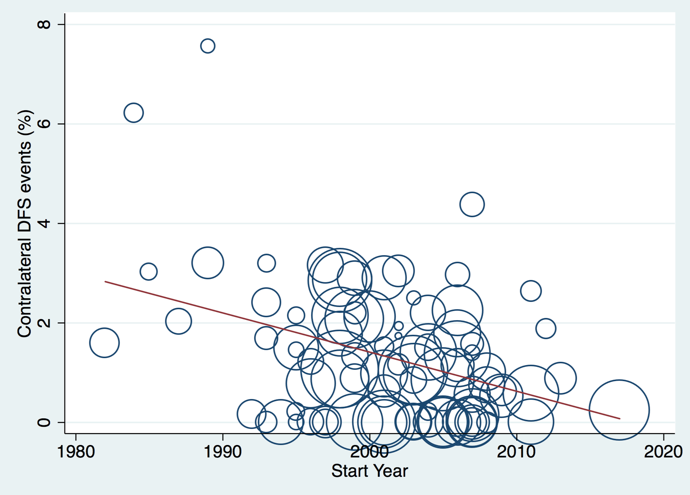


E. F.


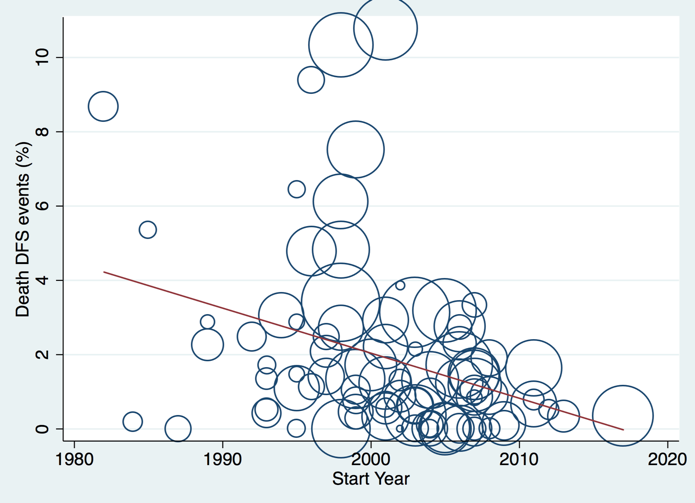

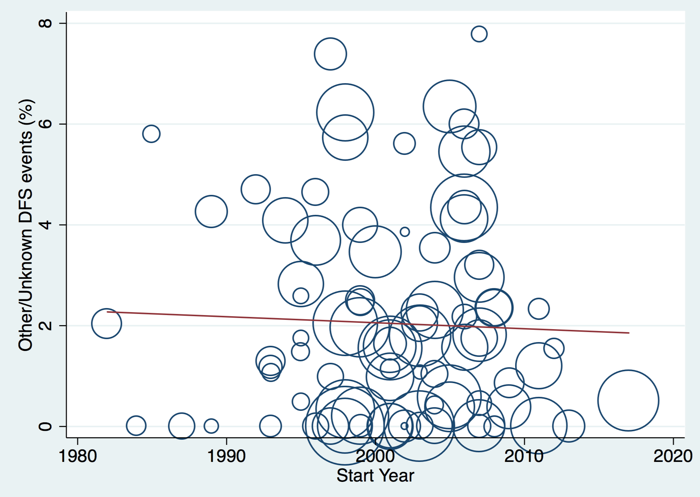


G. H.


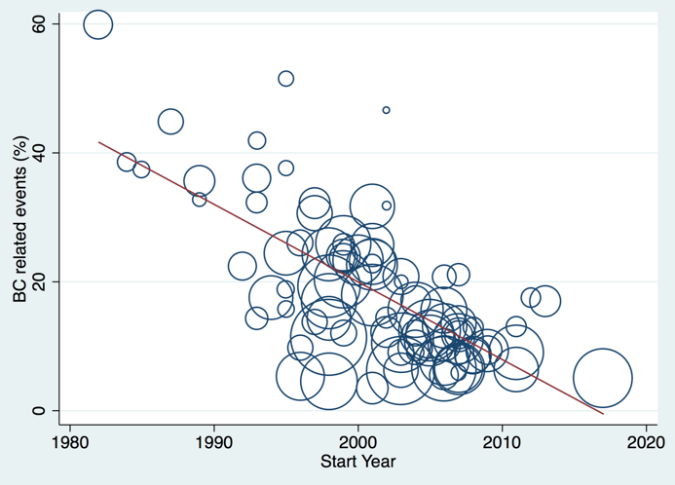

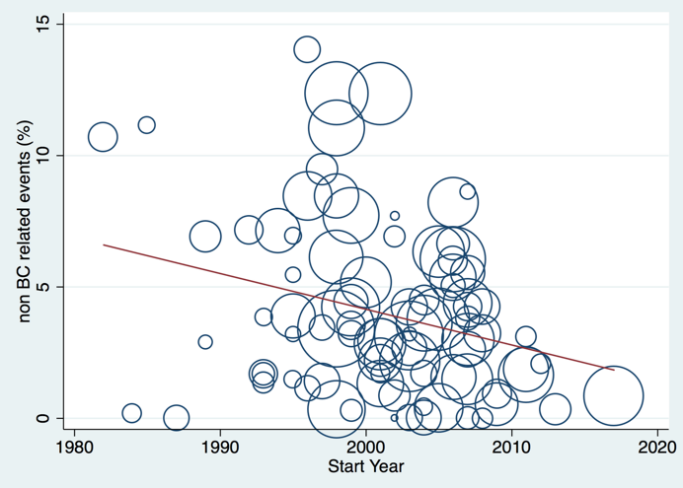


Supplemental Figures 3A-L: Analysis of DFS events as a proportion of all participants randomized (Column A) and as a proportion of DFS events (Column B) by follow-up duration (n=87 – 1 outlier excluded with FU time >300m)

A. B.


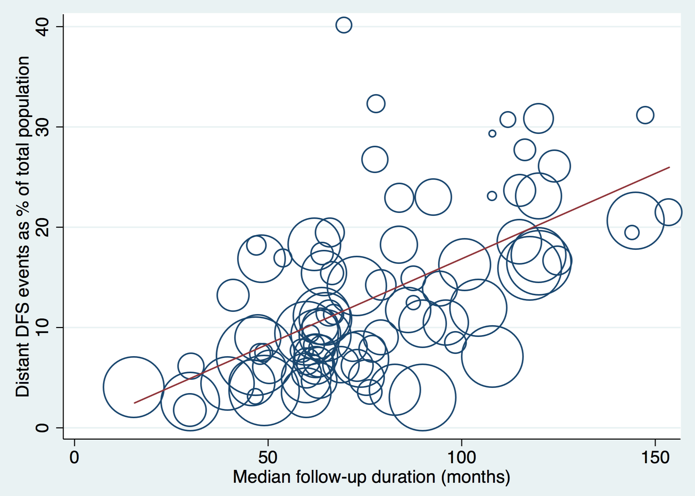

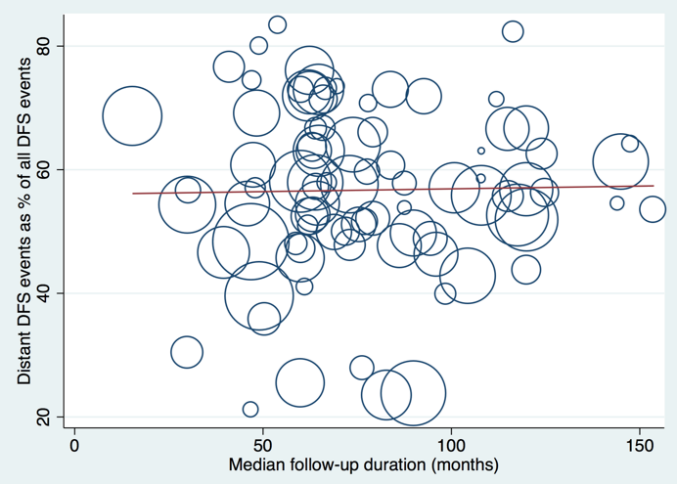


C. D.


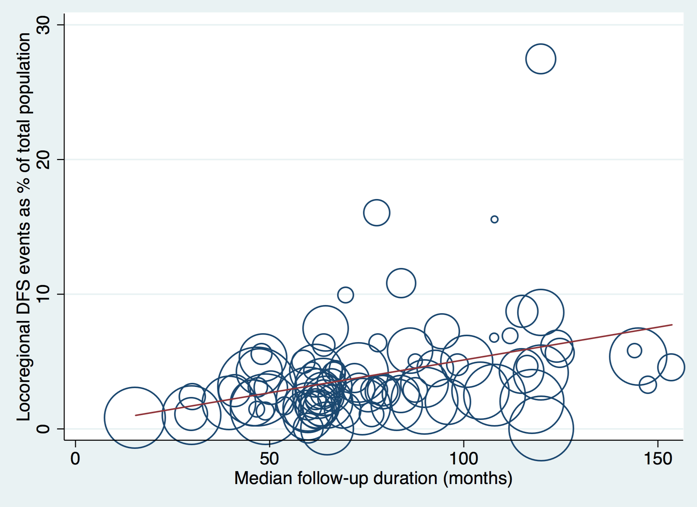

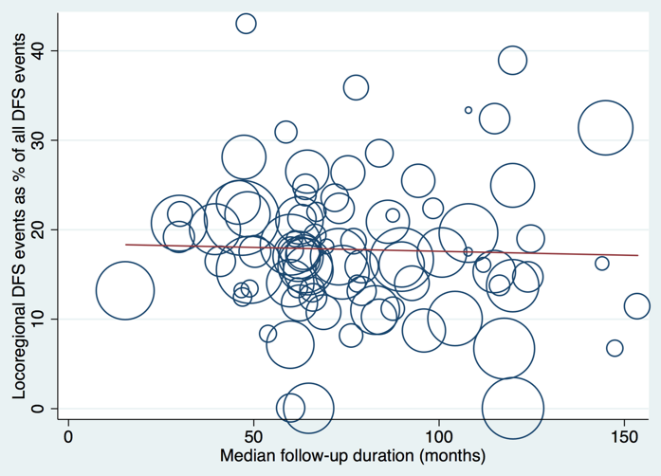


E. F.


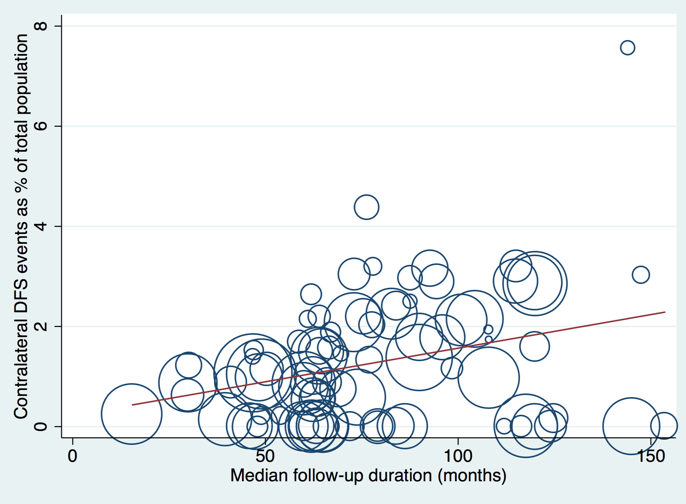

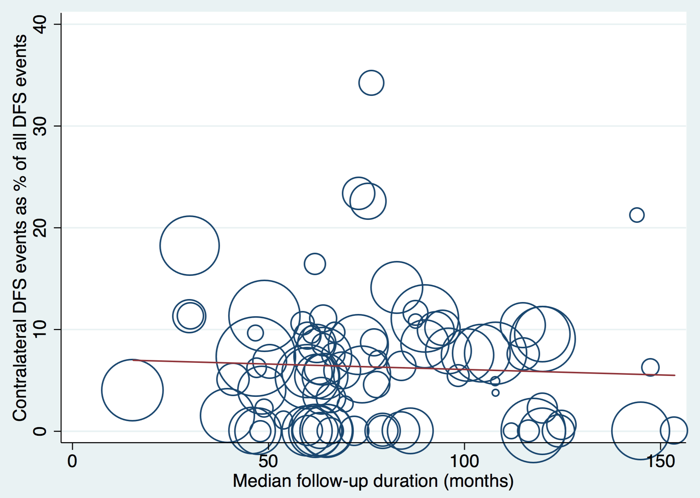


G. H.


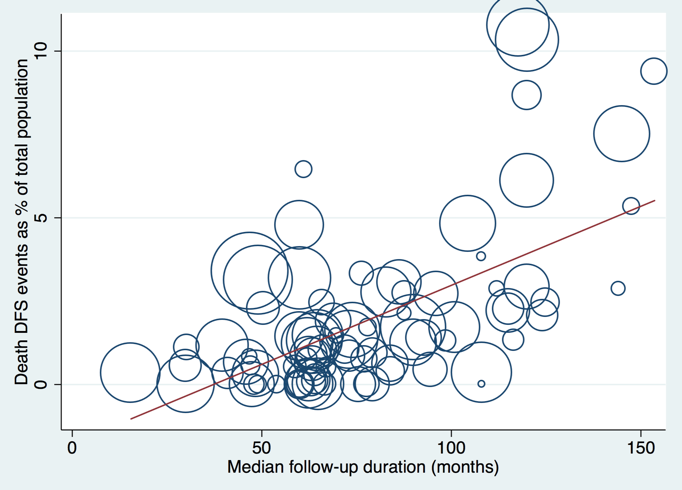

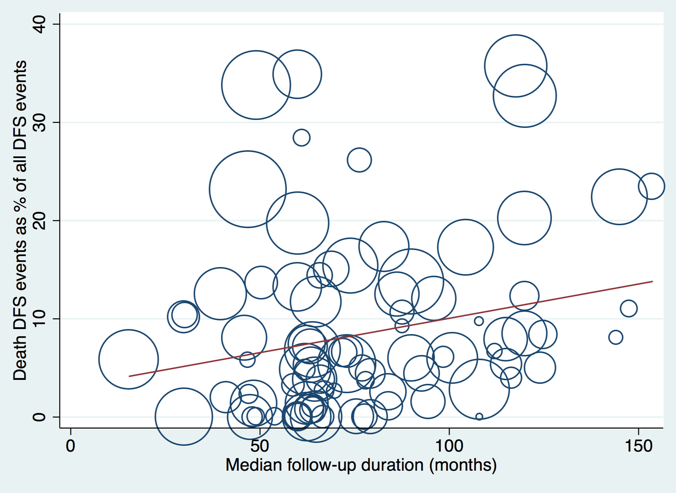


I. J.

**
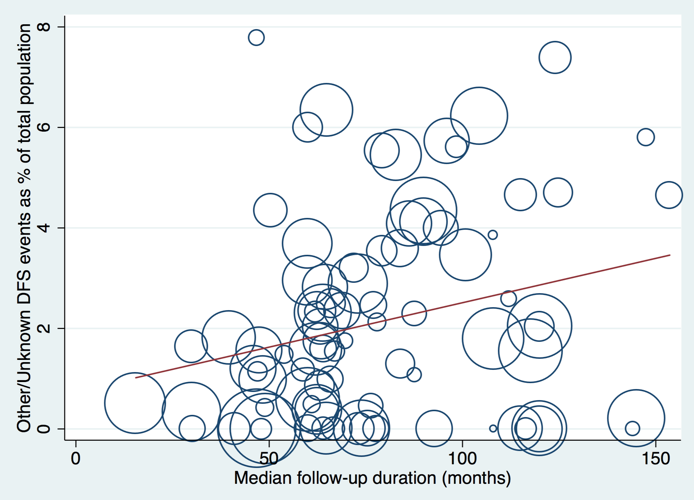

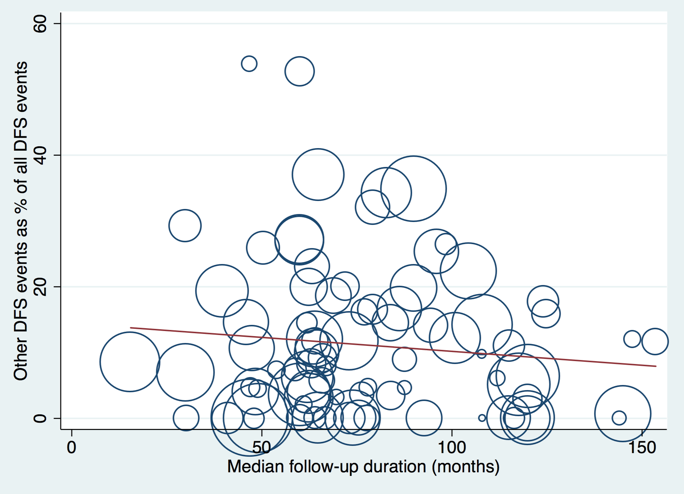
**

K. L.


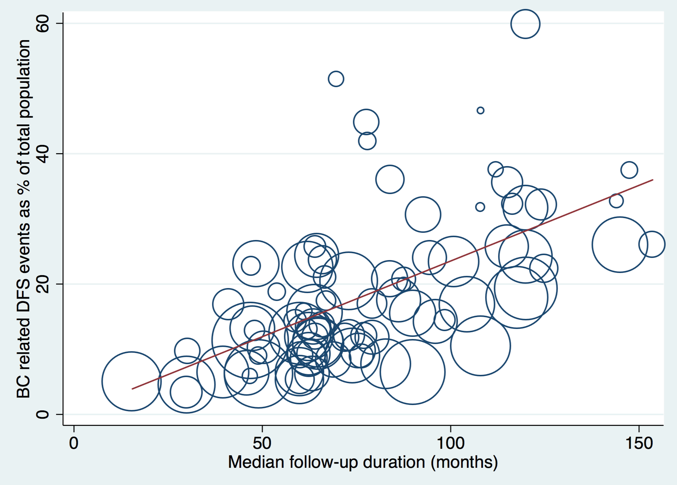

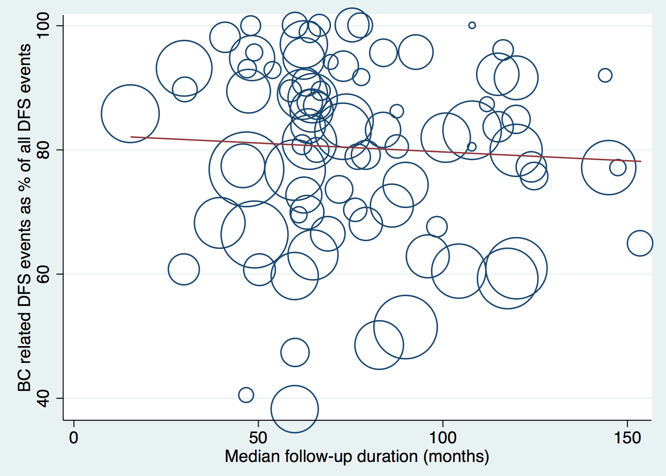


M. N.


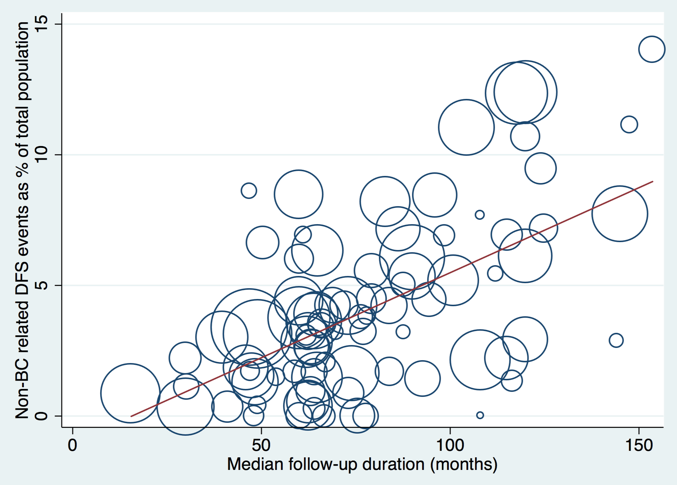

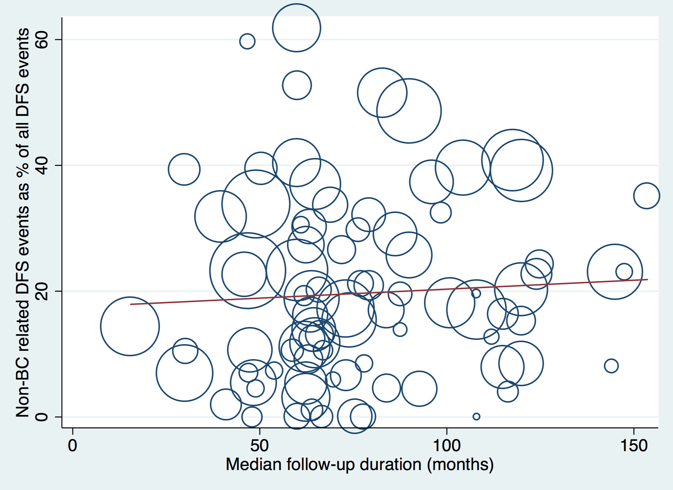


**Appendix 1: Search Strategy**

((breast cancer [Title/Abstract]) OR (breast neoplasia[Title/Abstract]) OR (breast malignancy[Title/Abstract])) AND ((adjuvant[Title/Abstract]) OR (early[Title/Abstract]) OR (stage I[Title/Abstract]) OR (stage II[Title/Abstract])OR (stage III[Title/Abstract])) AND ((phase 3) OR (phase three) OR (phase III))limit Clinical Trial or Randomized Controlled Trial

limit 01/01/2000 to 31/12/2020
